# 1-(2-Hy­droxy-3,5-dimeth­oxy­phen­yl)ethanone

**DOI:** 10.1107/S1600536811052974

**Published:** 2011-12-14

**Authors:** Wenming Li, Xiaobo Li, Yuqing Duan, Zhirong Deng, Runling Wang

**Affiliations:** a Tianjin Key Laboratory on Technologies Enabling Development of Clinical, Therapeutics and Diagnostics (Theranostics), School of Pharmacy, Tianjin Medical University, Tianjin 300070, People’s Republic of China

## Abstract

In title compound, C_10_H_12_O_4_, all of the non-H atoms lie approximately in a plane with the largest deviation being 0.061 (2) Å. An intra­molecular O—H⋯O hydrogen bond generates an *S*(6) ring motif. No classical inter­molecular hydrogen bonding occurs, with only van der Waals forces stabilizing the crystal structure.

## Related literature

For the biological activity of isoflavones, see: Wang & Murphy (1994 ▶); Yoshio *et al.* (1989 ▶). For bond-length data, see: Allen *et al.* (1987 ▶). For hydrogen-bond motifs, see: Bernstein *et al.* (1995 ▶). For the preparation, see: Aalten *et al.* (1989 ▶).
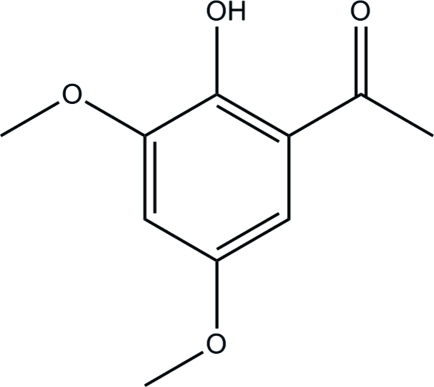


## Experimental

### 

#### Crystal data

C_10_H_12_O_4_
*M* *_r_* = 196.20
Monoclinic, 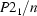

*a* = 7.733 (4) Å
*b* = 8.059 (4) Å
*c* = 14.851 (7) Å
β = 91.416 (10)°
*V* = 925.3 (7) Å^3^
*Z* = 4
Mo *K*α radiation
μ = 0.11 mm^−1^
*T* = 113 K
0.26 × 0.20 × 0.12 mm

#### Data collection

Rigaku Saturn724 CCD diffractometer
Absorption correction: multi-scan (*CrystalClear*; Rigaku/MSC, 2009 ▶) *T*_min_ = 0.972, *T*_max_ = 0.987
10288 measured reflections
2212 independent reflections
1621 reflections with *I* > 2σ(*I*)
*R*_int_ = 0.036

#### Refinement

*R*[*F* ^2^ > 2σ(*F* ^2^)] = 0.035
*wR*(*F* ^2^) = 0.091
*S* = 1.04
2212 reflections
131 parameters
H-atom parameters constrained
Δρ_max_ = 0.27 e Å^−3^
Δρ_min_ = −0.25 e Å^−3^

### 

Data collection: *CrystalClear-SM Expert* (Rigaku/MSC, 2009 ▶); cell refinement: *CrystalClear-SM Expert*; data reduction: *CrystalClear-SM Expert*; program(s) used to solve structure: *SHELXS97* (Sheldrick, 2008 ▶); program(s) used to refine structure: *SHELXL97* (Sheldrick, 2008 ▶); molecular graphics: *SHELXTL* (Sheldrick, 2008 ▶); software used to prepare material for publication: *SHELXTL*.

## Supplementary Material

Crystal structure: contains datablock(s) I, global. DOI: 10.1107/S1600536811052974/hg5147sup1.cif

Structure factors: contains datablock(s) I. DOI: 10.1107/S1600536811052974/hg5147Isup2.hkl

Supplementary material file. DOI: 10.1107/S1600536811052974/hg5147Isup3.cml

Additional supplementary materials:  crystallographic information; 3D view; checkCIF report

## Figures and Tables

**Table 1:** Hydrogen-bond geometry (Å, °)

*D*—H⋯*A*	*D*—H	H⋯*A*	*D*⋯*A*	*D*—H⋯*A*
O3—H3⋯O4	0.84	1.83	2.5666 (14)	145

**Fig. 1.**
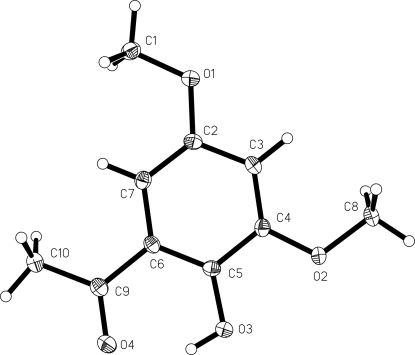
View of the title compound, with displacement ellipsoids drawn at the 40% probability level.

## supplementary crystallographic information

### Comment

Soy isoflavone is secondary metabolite during its growth period. As it could be extracted from plants and have a similar structure of estrogen, people usually call it phytoestrogen. Due to the manifested biological activity, such as antitumor, cardiovascular protection, anti-oxidant, anti-inflammatory, osteoporosis improvement, dual effect on estrogen, isoflavone has been paid more attention in social and academic area (Wang & Murphy, 1994; Yoshio *et al.*, 1989). During the development of our own isoflavone derivatives, the title compound, 1-(2-hydroxy-3,5-dimethoxyphenyl)ethanone, was prepared as an intermediate. The crystallographic analysis of the title compound described herein further confirms the molecular structures of the title compound and isoflavones.

In title compound, C_10_H_12_O_4_, all bond lengths and angles in the molecule are normal (Allen *et al.*, 1987). All of atoms (C1—C10/O1—O4, except H atoms)lie in a plane with the largest deviation 0.061 (2) Å for C10. The intramolecular O3—H3···O4 hydrogen bonds generate S(6) ring motif (Bernstein *et al.*, 1995). There is no classical intermolecular hydrogen bond found in the structure with only Van der Waals forces stabilizing the crystal.

### Experimental

Under ice bath, a solution of 2-hydroxyacetophenone(100 g, 0.734 mol) in CH_3_OH(1.2*L*) was added *N*-bromosuccinimide(392 g, 2.203 mol). Then the reaction mixture was stirred overnight at room temperature. The mixture was added 1*L* water to form yellow precipitation then filtered. The filtered cake was washed with a little amount of CH_3_OH/H_2_O=1/1 to yield 80 g light yellow crystals, which is 1-(3,5-dibromo-2-hydroxyphenyl)ethanone. Under ice bath, sodium methoxide(73 g, 1.360 mol) was dissolved in CH_3_OH (1*L*). Then under nitrogen protection, 1-(3,5-dibromo-2-hydroxyphenyl)ethanone (80 g, 0.272 mol) and CuCl(27 g,0.272 mol) was added to the solution quickly followed by DMF(0.5*L*). The brown suspension was heated to 363 K overnight until LC—MS showed complete. The mixture was neutralized with concentrated HCl to pH5–6, filtered through celite. Then it was extracted with ethyl acetate three times. The combined organic phase was washed with brine, dried over Na_2_SO_4_ and evaporated *in vacuo* to obtain crude product. Pure title compound was obtained by column chromatography. Crystals suitable for X-ray diffraction were obtained through slow evaporation of a solution of the pure title compound in ethyl acetate/n-hexane (1/4 by volume)(Aalten *et al.*, 1989).

### Refinement

All H atoms were found on difference maps, with C—H = 0.95 or 0.98, O—H = 0.84 Å and included in the final cycles of refinement using a riding model, with *U*_iso_(H) = 1.2*U*_eq_(C) and 1.5*U*_eq_(C, O) for the methyl and hydroxyl H atoms.

### Crystal data

**Table:**

C_10_H_12_O_4_	*F*(000) = 416
*M**_r_* = 196.20	*D*_x_ = 1.408 Mg m^−^^3^
Monoclinic, *P*2_1_/*n*	Mo *K*α radiation, λ = 0.71073 Å
Hall symbol: -P 2yn	Cell parameters from 3101 reflections
*a* = 7.733 (4) Å	θ = 1.4–27.9°
*b* = 8.059 (4) Å	µ = 0.11 mm^−^^1^
*c* = 14.851 (7) Å	*T* = 113 K
β = 91.416 (10)°	Prism, colorless
*V* = 925.3 (7) Å^3^	0.26 × 0.20 × 0.12 mm
*Z* = 4	

### Data collection

**Table:**

Rigaku Saturn724 CCD diffractometer	2212 independent reflections
Radiation source: rotating anode	1621 reflections with *I* > 2σ(*I*)
multilayer	*R*_int_ = 0.036
Detector resolution: 14.22 pixels mm^-1^	θ_max_ = 27.9°, θ_min_ = 2.7°
ω and φ scans	*h* = −10→10
Absorption correction: multi-scan (*CrystalClear*; Rigaku/MSC, 2009)	*k* = −10→10
*T*_min_ = 0.972, *T*_max_ = 0.987	*l* = −19→19
10288 measured reflections	

### Refinement

**Table:**

Refinement on *F*^2^	Primary atom site location: structure-invariant direct methods
Least-squares matrix: full	Secondary atom site location: difference Fourier map
*R*[*F*^2^ > 2σ(*F*^2^)] = 0.035	Hydrogen site location: inferred from neighbouring sites
*wR*(*F*^2^) = 0.091	H-atom parameters constrained
*S* = 1.04	*w* = 1/[σ^2^(*F*_o_^2^) + (0.0513*P*)^2^] where *P* = (*F*_o_^2^ + 2*F*_c_^2^)/3
2212 reflections	(Δ/σ)_max_ = 0.003
131 parameters	Δρ_max_ = 0.27 e Å^−^^3^
0 restraints	Δρ_min_ = −0.25 e Å^−^^3^

### Special details

**Table:**

Geometry. All e.s.d.'s (except the e.s.d. in the dihedral angle between two l.s. planes) are estimated using the full covariance matrix. The cell e.s.d.'s are taken into account individually in the estimation of e.s.d.'s in distances, angles and torsion angles; correlations between e.s.d.'s in cell parameters are only used when they are defined by crystal symmetry. An approximate (isotropic) treatment of cell e.s.d.'s is used for estimating e.s.d.'s involving l.s. planes.
Refinement. Refinement of *F*^2^ against ALL reflections. The weighted *R*-factor *wR* and goodness of fit *S* are based on *F*^2^, conventional *R*-factors *R* are based on *F*, with *F* set to zero for negative *F*^2^. The threshold expression of *F*^2^ > σ(*F*^2^) is used only for calculating *R*-factors(gt) *etc*. and is not relevant to the choice of reflections for refinement. *R*-factors based on *F*^2^ are statistically about twice as large as those based on *F*, and *R*- factors based on ALL data will be even larger.

### Fractional atomic coordinates and isotropic or equivalent isotropic displacement parameters (Å^2^)

**Table:**

	*x*	*y*	*z*	*U*_iso_*/*U*_eq_	
O1	0.91994 (10)	0.94416 (10)	0.30478 (5)	0.0250 (2)	
O2	1.34013 (9)	0.60797 (9)	0.45862 (5)	0.0214 (2)	
O3	1.15055 (10)	0.58744 (9)	0.60098 (5)	0.0211 (2)	
H3	1.0779	0.5817	0.6420	0.032*	
O4	0.86931 (10)	0.65890 (10)	0.68062 (5)	0.0244 (2)	
C1	0.75834 (15)	1.03005 (14)	0.30265 (8)	0.0231 (3)	
H1A	0.6636	0.9496	0.3057	0.035*	
H1B	0.7468	1.0937	0.2466	0.035*	
H1C	0.7537	1.1058	0.3542	0.035*	
C2	0.96176 (14)	0.85633 (13)	0.38174 (7)	0.0183 (2)	
C3	1.12493 (14)	0.77847 (12)	0.37976 (7)	0.0189 (2)	
H3A	1.1938	0.7880	0.3279	0.023*	
C4	1.18437 (14)	0.68864 (13)	0.45298 (7)	0.0175 (2)	
C5	1.08271 (13)	0.67472 (12)	0.53088 (7)	0.0169 (2)	
C6	0.92014 (13)	0.75150 (12)	0.53199 (7)	0.0171 (2)	
C7	0.85964 (14)	0.84275 (13)	0.45606 (7)	0.0185 (2)	
H7	0.7492	0.8943	0.4564	0.022*	
C8	1.44819 (15)	0.62000 (15)	0.38132 (8)	0.0248 (3)	
H8A	1.3885	0.5701	0.3290	0.037*	
H8B	1.5571	0.5611	0.3935	0.037*	
H8C	1.4724	0.7370	0.3688	0.037*	
C9	0.81400 (14)	0.73530 (13)	0.61314 (7)	0.0196 (2)	
C10	0.63652 (15)	0.81151 (15)	0.61438 (8)	0.0261 (3)	
H10A	0.5841	0.7892	0.6726	0.039*	
H10B	0.5642	0.7633	0.5660	0.039*	
H10C	0.6456	0.9316	0.6054	0.039*	

### Atomic displacement parameters (Å^2^)

**Table:**

	*U*^11^	*U*^22^	*U*^33^	*U*^12^	*U*^13^	*U*^23^
O1	0.0233 (4)	0.0318 (4)	0.0201 (4)	0.0054 (3)	0.0026 (3)	0.0080 (3)
O2	0.0161 (4)	0.0290 (4)	0.0192 (4)	0.0037 (3)	0.0035 (3)	0.0023 (3)
O3	0.0202 (4)	0.0268 (4)	0.0163 (4)	0.0024 (3)	0.0012 (3)	0.0028 (3)
O4	0.0248 (4)	0.0308 (5)	0.0177 (4)	0.0009 (3)	0.0024 (3)	0.0011 (3)
C1	0.0231 (6)	0.0236 (6)	0.0223 (6)	0.0024 (5)	−0.0021 (5)	0.0020 (5)
C2	0.0206 (6)	0.0183 (5)	0.0160 (5)	−0.0019 (4)	−0.0008 (4)	0.0005 (4)
C3	0.0192 (6)	0.0207 (5)	0.0170 (5)	−0.0025 (4)	0.0040 (4)	−0.0008 (4)
C4	0.0154 (5)	0.0181 (5)	0.0192 (5)	−0.0006 (4)	0.0010 (4)	−0.0029 (4)
C5	0.0196 (6)	0.0165 (5)	0.0146 (5)	−0.0017 (4)	−0.0013 (4)	−0.0012 (4)
C6	0.0185 (6)	0.0172 (5)	0.0158 (5)	−0.0020 (4)	0.0022 (4)	−0.0027 (4)
C7	0.0165 (5)	0.0186 (5)	0.0204 (6)	0.0000 (4)	−0.0003 (4)	−0.0020 (4)
C8	0.0189 (6)	0.0310 (6)	0.0249 (6)	0.0022 (5)	0.0081 (5)	0.0031 (5)
C9	0.0217 (6)	0.0191 (5)	0.0181 (5)	−0.0024 (4)	0.0012 (4)	−0.0034 (4)
C10	0.0238 (6)	0.0314 (6)	0.0234 (6)	0.0046 (5)	0.0060 (5)	0.0008 (5)

### Geometric parameters (Å, °)

**Table:**

O1—C2	1.3762 (14)	C3—H3A	0.9500
O1—C1	1.4283 (14)	C4—C5	1.4192 (15)
O2—C4	1.3695 (13)	C5—C6	1.4016 (15)
O2—C8	1.4398 (14)	C6—C7	1.4161 (16)
O3—C5	1.3516 (13)	C6—C9	1.4806 (16)
O3—H3	0.8400	C7—H7	0.9500
O4—C9	1.2430 (14)	C8—H8A	0.9800
C1—H1A	0.9800	C8—H8B	0.9800
C1—H1B	0.9800	C8—H8C	0.9800
C1—H1C	0.9800	C9—C10	1.5041 (16)
C2—C7	1.3773 (16)	C10—H10A	0.9800
C2—C3	1.4102 (16)	C10—H10B	0.9800
C3—C4	1.3758 (16)	C10—H10C	0.9800
			
C2—O1—C1	117.07 (9)	C5—C6—C7	119.91 (9)
C4—O2—C8	116.50 (9)	C5—C6—C9	119.08 (10)
C5—O3—H3	109.5	C7—C6—C9	121.00 (10)
O1—C1—H1A	109.5	C2—C7—C6	119.65 (10)
O1—C1—H1B	109.5	C2—C7—H7	120.2
H1A—C1—H1B	109.5	C6—C7—H7	120.2
O1—C1—H1C	109.5	O2—C8—H8A	109.5
H1A—C1—H1C	109.5	O2—C8—H8B	109.5
H1B—C1—H1C	109.5	H8A—C8—H8B	109.5
O1—C2—C7	125.37 (10)	O2—C8—H8C	109.5
O1—C2—C3	113.80 (9)	H8A—C8—H8C	109.5
C7—C2—C3	120.83 (10)	H8B—C8—H8C	109.5
C4—C3—C2	119.95 (10)	O4—C9—C6	120.88 (11)
C4—C3—H3A	120.0	O4—C9—C10	119.23 (10)
C2—C3—H3A	120.0	C6—C9—C10	119.89 (10)
O2—C4—C3	125.05 (9)	C9—C10—H10A	109.5
O2—C4—C5	114.62 (9)	C9—C10—H10B	109.5
C3—C4—C5	120.32 (10)	H10A—C10—H10B	109.5
O3—C5—C6	123.49 (9)	C9—C10—H10C	109.5
O3—C5—C4	117.18 (10)	H10A—C10—H10C	109.5
C6—C5—C4	119.33 (10)	H10B—C10—H10C	109.5
			
C1—O1—C2—C7	0.96 (15)	O3—C5—C6—C7	179.09 (9)
C1—O1—C2—C3	−178.46 (9)	C4—C5—C6—C7	−0.45 (15)
O1—C2—C3—C4	178.95 (9)	O3—C5—C6—C9	−1.01 (15)
C7—C2—C3—C4	−0.50 (16)	C4—C5—C6—C9	179.46 (9)
C8—O2—C4—C3	0.15 (15)	O1—C2—C7—C6	−178.46 (10)
C8—O2—C4—C5	−179.49 (9)	C3—C2—C7—C6	0.92 (16)
C2—C3—C4—O2	179.97 (9)	C5—C6—C7—C2	−0.44 (16)
C2—C3—C4—C5	−0.41 (16)	C9—C6—C7—C2	179.66 (9)
O2—C4—C5—O3	0.96 (14)	C5—C6—C9—O4	2.04 (15)
C3—C4—C5—O3	−178.69 (9)	C7—C6—C9—O4	−178.05 (10)
O2—C4—C5—C6	−179.47 (9)	C5—C6—C9—C10	−177.77 (10)
C3—C4—C5—C6	0.87 (15)	C7—C6—C9—C10	2.13 (15)

### Hydrogen-bond geometry (Å, °)

**Table:**

*D*—H···*A*	*D*—H	H···*A*	*D*···*A*	*D*—H···*A*
O3—H3···O4	0.84	1.83	2.5666 (14)	145.
